# No UCP1 in the kidney

**DOI:** 10.1016/j.molmet.2025.102127

**Published:** 2025-03-20

**Authors:** Celso Pereira Batista Sousa-Filho, Natasa Petrovic

**Affiliations:** Department of Molecular Biosciences, The Wenner-Gren Institute, Stockholm University, SE-106 91 Stockholm, Sweden

**Keywords:** UCP1, Brown adipose tissue, Kidney, Immunohistochemistry, Antibody

## Abstract

**Objectives:**

Several recent studies have indicated the presence of UCP1 in the kidney, challenging the paradigm that UCP1 is only found in brown and beige adipocytes and broadening the (patho)physiological significance of UCP1. The kidney localization has been the direct result of immunohistochemical investigations and an inferred outcome from multiple lines of reporter mice. These findings require confirmation and further physiological characterization.

**Methods:**

We examined UCP1 expression in the kidney using immunohistochemistry and qPCR. Transversal sections through or near the kidney hilum, consistently including perirenal brown fat and adjacent kidney tissue, were analyzed with four UCP1 antibodies.

**Results:**

In addition to detecting UCP1 in perirenal adipose tissue, we observed distinct immunopositive structures in the kidney with our in-house UCP1-antibody, ‘C10’, in apparent agreement with earlier reports. To corroborate this, we tested the C10-antibody on kidney sections from UCP1-ablated mice but found equal reactivity in these UCP1-negative tissues. We then tested the widely used antibody ab10983, previously employed in kidney studies. Also here, the positive signal persisted in UCP1-ablated mice, clearly invalidating earlier findings. UCP1 qPCR studies also failed to detect UCP1 mRNA above background. Finally, two highly specific antibodies, E9Z2V and EPR20381, accurately detected UCP1 in perirenal adipose tissue but showed no signal in the kidney.

**Conclusions:**

When appropriate controls are implemented, there is no evidence for the presence of UCP1 in the kidney. Consequently, this conclusion also implies that the results from UCP1 reporter mice, specifically regarding kidney expression of the UCP1 gene – though possibly applicable to other tissues – require reconfirmation before being accepted as evidence for the presence of UCP1 in non-adipose tissues.

## Highlights

• To validate the reported UCP1 presence in the kidney, we examined its expression in this organ.
• Immunohistochemical analysis showed no evidence for UCP1 in the kidney.
• qPCR analysis detected no Ucp1 mRNA in the kidney.
• UCP1 protein is not present even in minor subpopulations of kidney cells.
• Our data reconfirm the unique localization of UCP1 in adipose tissues.

## Graphical abstract

**Figure:**
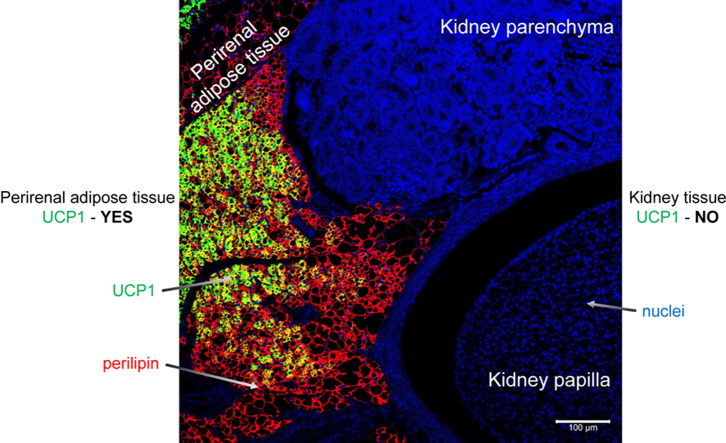

## 1 Introduction

Uncoupling protein 1 (UCP1) is considered to be highly specific to brown [1] and beige (e.g. [2]) adipocytes. However, UCP1 mRNA and/or UCP1 protein have repeatedly been reported in tissues other than brown and beige fat (as detailed in the Discussion), including studies suggesting that UCP1 is expressed in the kidney. Thus, in studies using Ucp1-Cre mouse models [3,4], Cre-mediated labelling of cells (that should reflect Ucp1 promoter activity and thus UCP1 expression) has been observed in the kidney [[5], [6], [7]]. Additionally, based on immunoblotting and immunostaining analyses, a series of studies have directly reported the presence of UCP1 protein in the kidney [[8], [9], [10], [11], [12]]. These studies have also suggested (patho)physiological roles for UCP1 in kidney function, such as the induction of a catabolic state known as ‘tumor slimming’ [9,10], defence against oxidative stress [8,12], and alleviation of lipid accumulation [11].

In a study investigating the contribution of different brown fat depots to the nonshivering thermogenic capacity in mice, we have analysed several adipose tissue depots, including perirenal brown adipose tissue (perirenal BAT). During histological analysis of perirenal BAT using our in-house UCP1 antibody, we also observed intense, distinct staining for UCP1 in the adjacent kidney tissue. This noteworthy finding was thus consistent with the published reports mentioned above, and it piqued our interest in this phenomenon. However, given the kidney's exceptionally high energy demands, the energy-dissipating function of UCP1 in this organ seemed paradoxical. This raised the possibility that UCP1, if present, may function differently – either not as an uncoupler or as an uncoupler with low activity – and could play a critical yet underexplored physiological role. We therefore found it necessary to rigorously evaluate the presence of UCP1 in the kidney.

## 2 Experimental procedures

### 2.1 Animals

All experiments were approved by the Animal Ethics Committee of the North Stockholm region. Before the start of the experiments, all mice (both sexes) were housed at 21–24 °C in a 12:12-h light–dark cycle, with free access to chow food (Brogaarden Altromin 1324) and water.

### 2.2 Cold acclimation

Female C57Bl/6 mice, bred at the institute, remained in their original cages until approximately 6 weeks of age. The mice were then single-caged and transferred to 18 °C for 1–2 weeks (according to the ethical permit), followed by transfer to 4 °C for the subsequent 8 weeks.

### 2.3 UCP1–KO mice

UCP1-ablated mice were progeny of those described in [13], backcrossed to the C57Bl/6J background (Jackson Laboratory strain 003124). The mice were bred and maintained in-house as homozygous lines (UCP1-knockout and wild-type). To avoid genetic drift, UCP1-knockout and wild-type lines were regularly intercrossed. The mice remained in their original cages until they were sacrificed at approximately 8 weeks of age.

### 2.4 Sampling of tissues

At the indicated times (the end of the experiments), the animals were sacrificed using CO_2_ anaesthesia. The right kidney, along with the perirenal brown adipose tissue (perirenal BAT), was placed in a formaldehyde solution and used for immunohistochemical analysis. The left kidney, with all visible adipose tissue removed, was snap-frozen in liquid nitrogen and used for qPCR analysis. Interscapular brown adipose tissue (IBAT), inguinal white adipose tissue (ingWAT) and gonadal white adipose tissue (gWAT) were quantitatively dissected, snap-frozen in liquid nitrogen and stored at −80 °C. A liver sample, intended for qPCR analysis, was also in liquid nitrogen.

### 2.5 Immunohistochemistry

Immunohistochemistry was performed principally as in [14]. A detailed description of the immunohistological analyses is provided in the Supplement.

### 2.6 Gene expression analysis

RNA isolation, cDNA synthesis and real-time qPCR were performed as in [14] (see the Supplement for details).

## 3 Results

Our observation of intense UCP1 immunoreactivity in the kidney, along with the earlier reports indicating UCP1 expression in this organ [[5], [6], [7], [8], [9], [10], [11], [12]], motivated us to investigate whether UCP1 is indeed expressed in kidney tissue. We have here rigorously evaluated its presence in the kidney.

### 3.1 UCP1 protein is apparently expressed in the kidney of mice acclimated to 4 °C

Perirenal BAT is a small visceral adipose depot located at the hilum of the kidney [15,16]. This brown fat depot exhibits remarkable plasticity during postnatal life and in response to environmental temperature changes [16,17]. Among brown fat depots, perirenal BAT shows the greatest relative increase (∼100-fold) in UCP1 mRNA level upon cold acclimation [16]. For this reason, we elected to use tissue from cold-acclimated mice for a histological examination of perirenal BAT.

**Figure 1:**
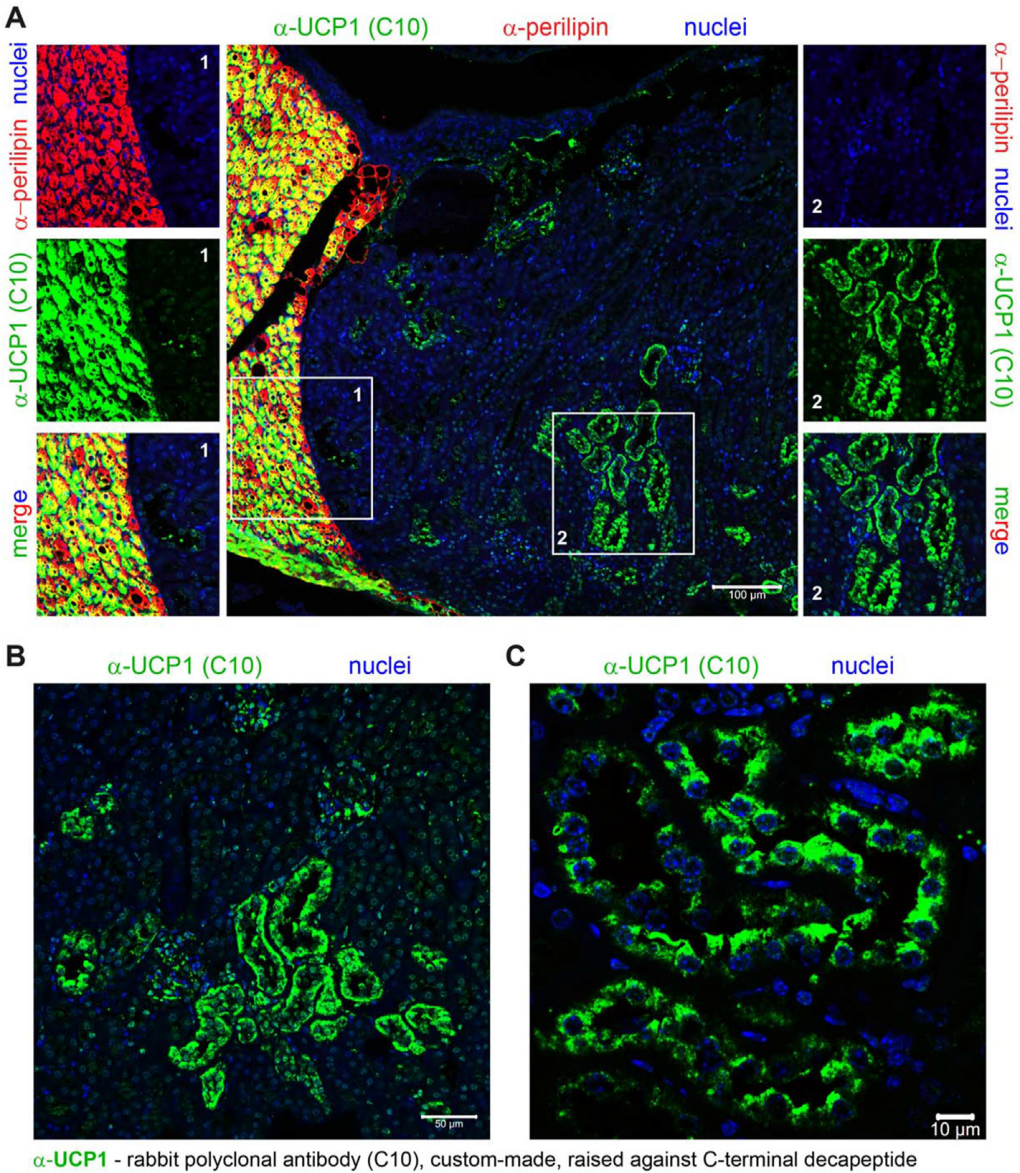
**Immunoreactivity to the UCP1 antibody C10 in the perirenal brown adipose tissue (perirenal BAT) and the kidney of mice acclimated to 4 °C. (A**–**C)** Representative confocal images obtained using the rabbit polyclonal antibody raised against the C-terminal decapeptide of UCP1 (C10). **(A)** Representative confocal image of the transversal section of the kidney, also showing adjacent perirenal BAT. UCP1 – green; perilipin (an adipocyte identity marker) – red; nuclei – blue. Magnified insets provide better visualization of perirenal BAT (inset 1) and the kidney parenchyma (inset 2). Scale bar, 100 μm. **(B)** and **(C)** Representative confocal images of the transversal section of the kidney acquired with higher magnification. UCP1 – green; nuclei – blue; note the absence of immunoreactivity for perilipin. Scale bar 50 μm (B) and 10 μm (C). (For interpretation of the references to color/colour in this figure legend, the reader is referred to the Web version of this article.)

Given that perirenal BAT is a very small adipose depot (approximately 10 mg), for practical reasons, it was dissected, processed and sectioned together with the adjacent kidney. Paraffin-embedded tissues were sectioned transversely through or near the kidney hilum. Adipocytes were visualized by staining for the lipid-droplet protein perilipin (red) (Figure 1A–C). The staining was intense in the perirenal adipose tissue, but no perilipin-positive cells were detected in the kidney (Figure 1A–C). To determine whether these perilipin-positive fat cells were brown adipocytes, we used our custom-made UCP1 antibody, raised against the C-terminal decapeptide (here referred to as the “C10 antibody”) (green) (Figure 1A). As shown in the main Figure 1A and the magnified inset 1, nearly all perilipin-stained cells (red) were also strongly positive for UCP1 (green), resulting in a yellow signal in the merged images (some clearly double-stained cells did not appear yellow due to the predominant red or green staining, causing them to appear primarily red or green in the overlays). Thus, perirenal BAT in these cold-acclimated mice was essentially composed solely of brown adipocytes (in agreement with [15,17]).

However, this staining with the UCP1 antibody also produced a strong, distinct signal (green) within the kidney itself (main Figure 1A and magnified inset 2). Images acquired at higher magnification (Figure 1B,C) provided greater detail of the morphology of these labelled structures. They were localized to specific regions of the kidney, most likely within tubular epithelial cells. Notably, the appearance of these structures resembled those detected in the kidney with the commercially available Abcam antibody (ab10983) [[8], [9], [10], [11], [12]]. These results may therefore be considered to indicate that UCP1 protein is expressed in the kidney of cold-acclimated mice.

### 3.2 The apparent expression of UCP1 in the kidney is probably attributable to antibody cross-reactivity

The apparent UCP1 expression in the kidney could either reflect actual UCP1 expression in this tissue or result from the antibody binding to a different protein (antibody cross-reactivity). To evaluate the nature of the signal obtained with the UCP1 antibody C10, we used mice with global UCP1 ablation (UCP1–KO mice) [13]. The complete lack of UCP1 protein in these animals would enable us to establish whether the signal observed in the kidney originates from the antibody binding specifically to UCP1 or from nonspecific binding to another protein. By consistently using sections that included both the perirenal BAT and the adjacent kidney, we ensured an integrated positive control for each staining series.

**Figure 2:**
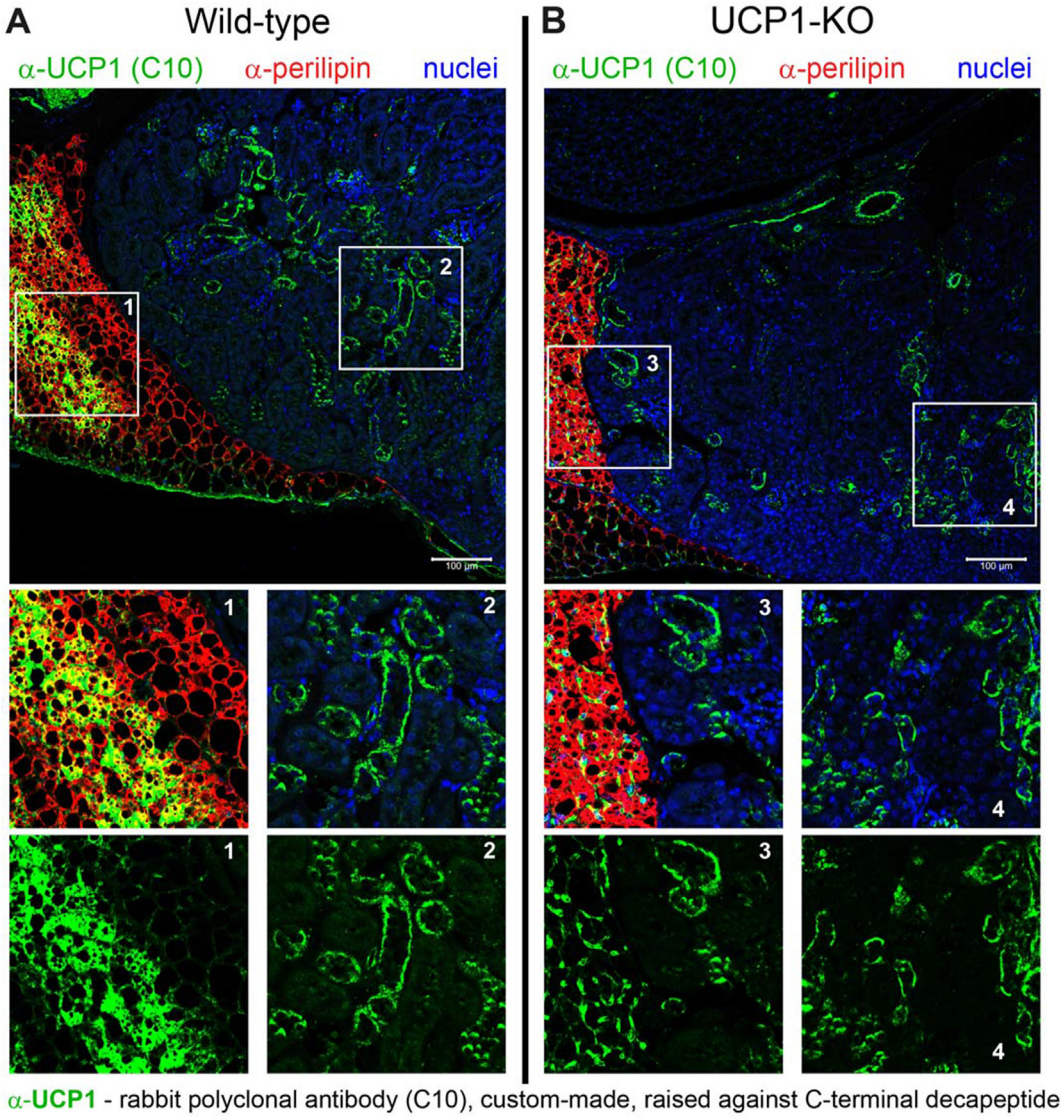
**Immunoreactivity to the UCP1 antibody in the kidney of UCP1-deficient mice suggests antibody cross-reactivity. (A**–**B)** Representative confocal images of the transversal section of the kidney, also showing adjacent perirenal BAT, obtained using the C10 UCP1 antibody. Wild-type (A) and UCP1–KO (B) mice, acclimated to room temperature and approximately two months old, were analysed. UCP1 – green; perilipin (an adipocyte identity marker) – red; nuclei – blue. Magnified insets provide better visualization of perirenal BAT (insets 1 and 3) and the kidney parenchyma (insets 2 and 4). Scale bars, 100 μm. (For interpretation of the references to color/colour in this figure legend, the reader is referred to the Web version of this article.)

The sections analysed here were from young male wild-type and UCP1-knockout mice acclimated to standard animal house conditions (∼21–22 °C). Again, in the perirenal BAT of the wild-type mice, UCP1 immunopositivity (green) was detected in a substantial portion of adipocytes (stained red), identifying them as brown adipocytes (appearing yellow in the overlay images) (main Figure 2A and magnified inset 1) (consistent with [17]). In the kidney of the wild-type mice, the UCP1 antibody C10 also produced a strong signal (main Figure 2A and magnified inset 2), resembling the signal observed in animals acclimated to cold (Figure 1). However, remarkably, very similar staining was observed in sections from UCP1–KO mice (main Figure 2B and magnified insets 3 and 4). This strongly indicates that the immunopositivity observed in the kidney results from nonspecific binding of the C10 antibody.

It may additionally be noted that off-target staining of another type was observed in the perirenal BAT of UCP1–KO mice (main Figure 2B and magnified inset 3). As expected, the adipocytes themselves (stained red) were negative for UCP1. However, elongated tubular structures between the adipocytes were strongly labelled with the UCP1 antibody (green). These structures probably represent blood vessels that are abundantly present in adipose tissue [18].

It is important to note that the appearance of these off-target structures in both the kidney and the perirenal BAT of the UCP1–KO mice is influenced by the immunohistochemistry protocol used (as explained in the legend for Figure S1). When a modified staining protocol with less-sensitive signal detection was applied, the only immunopositivity observed was in the perirenal BAT of wild-type mice ( Figure S1A and magnified inset 2 in that figure), with virtually no staining in the kidney (Figure S1A and magnified insets 1 and 3) or in the perirenal BAT of UCP1–KO mice (Figure S1B and magnified inset 4). This reinforces the conclusion that the staining initially observed in the kidney was presumably due to nonspecific binding of the UCP1 antibody, which became visible when using a very sensitive protocol.

### 3.3 Immunopositivity observed with a commercial UCP1 antibody in the kidneys of UCP1-deficient mice further indicates that UCP1 protein is not expressed in the kidney

The apparent staining for UCP1 in the kidney reported earlier was observed with the commercially available and widely used UCP1 antibody ab10983 from Abcam [[8], [9], [10], [11], [12]]. According to Abcam, this antibody has more than 800 citations. However, since the absence of cross-reactivity for this antibody was not explicitly demonstrated in those studies, we also assessed this antibody here, using similar samples from wild-type and UCP1–KO mice as those used for the C10 antibody.

**Figure 3:**
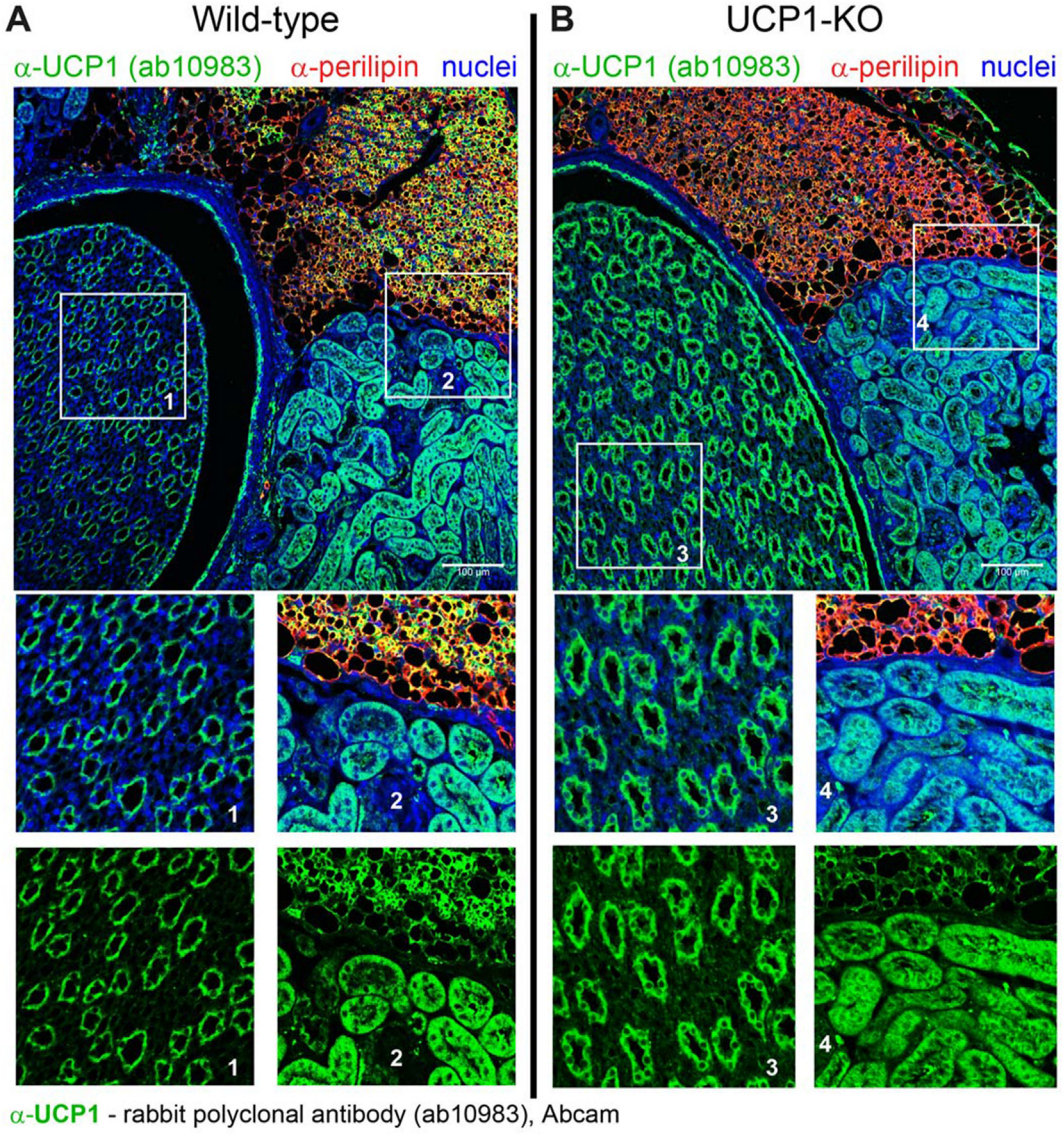
**Immunoreactivity to the commercial UCP1 antibody ab10983 in the kidney of UCP1-deficient mice further suggests that the staining is not due to UCP1. (A**–**B)** Representative confocal images of the transversal section of the kidney, also showing adjacent perirenal BAT, obtained using the rabbit polyclonal antibody from Abcam (ab10983). Wild-type (A) and UCP1–KO (B) mice, acclimated to room temperature and approximately two months old, were analysed. UCP1 – green; nuclei – blue. Magnified insets provide better visualization of the kidney papilla (insets 1 and 3) and the kidney parenchyma (insets 2 and 4). Scale bars, 100 μm. (For interpretation of the references to color/colour in this figure legend, the reader is referred to the Web version of this article.)

As shown in Figure 3A, the UCP1 antibody ab10983 resulted, as expected, in strong staining (green) of a substantial portion of adipocytes (labelled red) in the perirenal BAT of wild-type mice (main Figure 3A and magnified inset 2). However, weaker but still clearly detectable staining was observed in the perirenal BAT of UCP1–KO mice (main Figure 3B and magnified inset 4). This staining suggests that the antibody ab10983 is not UCP1-specific, even in brown adipose tissue. Importantly, in the kidney of wild-type mice, this antibody detected distinct structures in both the parenchyma and papilla (Figure 3A and magnified insets 1 and 2). These structures were intensely stained, easily distinguishable, and closely resembled those reported previously with this antibody [[8], [9], [10], [11], [12]]. Notably, the staining pattern in the kidney of UCP1–KO mice (Figure 3B and magnified insets 3 and 4) was virtually identical to that in wild-type mice, both in intensity and appearance (Figure 3B vs. Figure 3A). Thus, the ab10983 antibody displayed cross-reactivity in the kidney, resulting in a signal that cannot be attributed to UCP1 itself. The appearance of these off-target structures in the kidney was principally unaffected by the use of a less sensitive detection method (Figure S2), where they were merely less intense and somewhat more difficult to discern.

In summary, both antibodies that showed apparent UCP1 staining in the kidney (C10 and ab10983) produced this staining due to cross-reactivity. As a result, the signals detected with these antibodies cannot be attributed to the presence of UCP1 in the kidney.

### 3.4 No detectable UCP1 mRNA in the kidney

Although no immunohistochemical evidence supported UCP1 expression in the kidney, this could not exclude the theoretical possibility that UCP1 could be expressed at such low levels that it remained undetectable by immunohistochemistry (Figure S1), particularly in a manner distinguishable from the nonspecific staining observed with signal amplification (Figure 1, Figure 2, Figure 3). To address this possibility, we measured UCP1 mRNA levels in the kidney using qPCR. Liver, that does not express UCP1, served as a negative control. IBAT served as a positive control, while inguinal WAT (ingWAT), known to express very low levels of UCP1 under standard conditions, was included to verify method sensitivity. Gonadal WAT (gWAT), that essentially does not express UCP1 under standard conditions, was also included as a negative control. Tissues from UCP1–KO mice provided an additional negative control.

**Figure 4:**
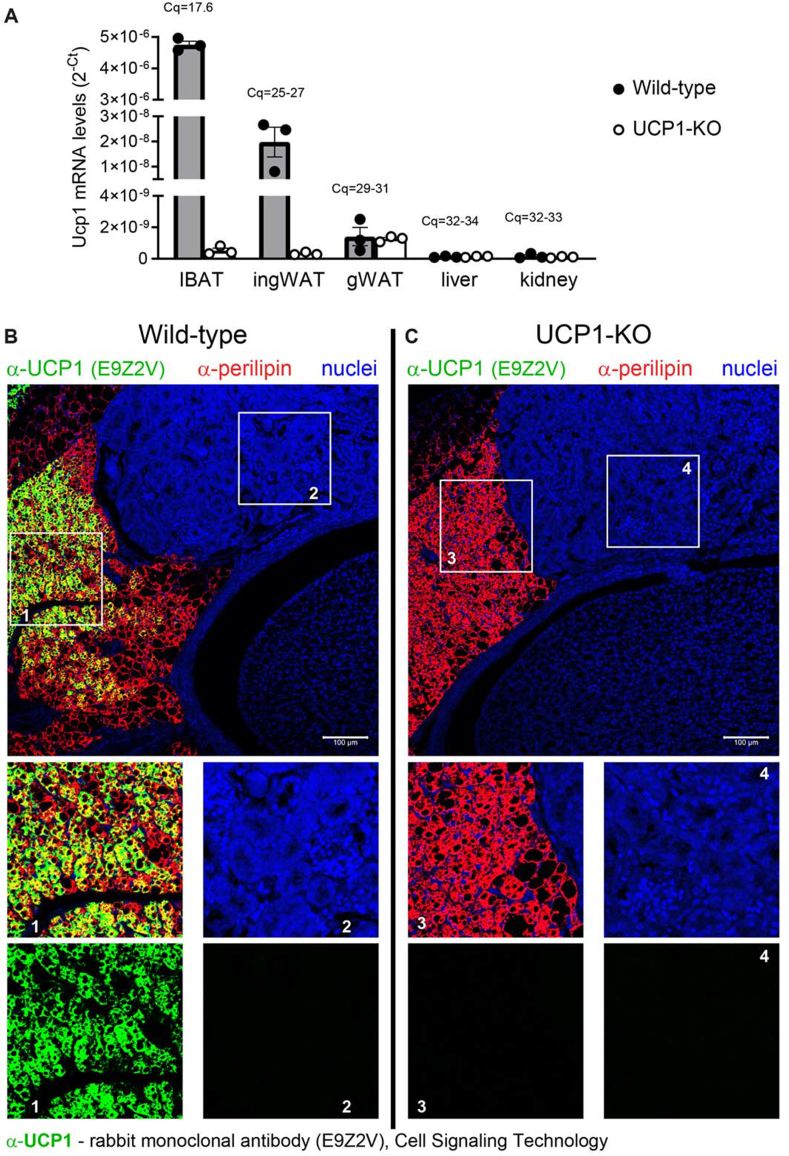
**UCP1 is not expressed in the kidney. (A)** UCP1 mRNA levels in IBAT (interscapular BAT), ingWAT (inguinal WAT), gWAT (gonadal WAT), liver and kidney of wild-type (n = 3) and UCP1–KO (n = 3) mice acclimated to room temperature and approximately two months old. Each symbol represents a sample from one mouse. Values are antilog-transformed Cq values (2^−Cq^) presented as means ± SEM. For each tissue, the mean Cq value is reported. Higher Cq values thus indicate lower expression levels. **(B–C)** Representative confocal images of the transversal section of the kidney, also showing adjacent perirenal BAT, obtained using the rabbit monoclonal antibody (E9Z2V) from Cell Signaling Technology (72298S). Wild-type (B) and UCP1–KO (C) male mice, acclimated to room temperature and approximately two months old, were analysed. UCP1 – green; perilipin (an adipocyte identity marker) – red; nuclei – blue. Note the complete absence of immunoreactivity to this UCP1 antibody in both the kidney and the perirenal BAT of UCP1-deficient mice (C). Magnified insets provide better visualization of perirenal BAT (insets 1 and 3) and the kidney parenchyma (insets 2 and 4). Scale bars, 100 μm. (For interpretation of the references to color/colour in this figure legend, the reader is referred to the Web version of this article.)

Among the analysed tissues, only IBAT from wild-type mice exhibited high levels of UCP1 expression (Figure 4A). UCP1 was clearly expressed in inguinal WAT, but UCP1 mRNA levels in this tissue were about 250 times lower than in IBAT. Thus, the method demonstrated a broad linear dynamic range and high sensitivity. Importantly, UCP1 mRNA was essentially undetectable in gonadal WAT and liver from wild-type mice, which served as negative controls. Cq values for amplified products in these tissues were above 30, presumably indicating nonspecific products, as also observed in tissues from UCP1–KO mice (Figure 4A, open symbols). Similarly, qPCR analysis of UCP1 gene expression in the kidney showed results comparable to those in the liver, with Cq values exceeding 30. Therefore, it can be concluded that the UCP1 gene is not expressed in the kidney.

### 3.5 UCP1 is not present even in minor kidney cell subpopulations

Although all the results presented in this study strongly suggest that UCP1 protein is not expressed in the kidney, the possibility remained that UCP1 might be expressed in a small fraction of cells too sparsely distributed to be detected through bulk qPCR analysis and potentially overlooked due to antibody cross-reactivity. To address this, the analysis was expanded by employing two additional commercially available antibodies, which, according to the manufacturers, were expected to exhibit satisfactory specificity.

The antibody from Cell Signaling (E9Z2V) was thoroughly validated by the manufacturer that reported that it detected UCP1 exclusively in BAT among the analysed tissues (colon, liver, lung, ovary, and pancreas). The kidney was thus not included in this validation. Also in our experiments, this antibody demonstrated extraordinary specificity: a signal was detected only in the perirenal BAT of wild-type mice (main Figure 4B and magnified inset 1). No signal was observed in the perirenal BAT of UCP1–KO mice (main Figure 4C and magnified inset 3) or in the kidney of either genotype (Figure 4BC and magnified insets 2 and 4), even though the UCP1 antibody-derived signal was detected with high sensitivity.

The antibody from Abcam (EPR20381), tested by the manufacturer in brown versus white fat, displayed high specificity for brown fat. In our experiments, this antibody also demonstrated excellent specificity: a signal was detected only in the perirenal BAT of wild-type mice (Figure S3A and magnified inset 1 in that figure), with no signal observed in the perirenal BAT of UCP1–KO mice (Figure S3B and magnified inset 3) or in the kidneys of either genotype (Figure S3AB, magnified insets 2 and 4). The UCP1 antibody-derived signal was again here detected using a highly sensitive detection method.

Thus, despite employing a highly sensitive detection method, both UCP1 antibodies (E9Z2V and EPR20381) demonstrated exceptional specificity, showing no cross-reactivity in the kidney. Even with these highly specific antibodies, there was no evidence of any kidney cell population genuinely expressing UCP1.

In summary, all the data presented in this study, including highly sensitive qPCR analysis and thorough immunohistochemical analysis with four different antibodies, failed to provide any evidence that UCP1 protein is found in the kidney.

## 4 Discussion

### 4.1 Observing UCP1 outside brown (and beige) adipocytes – a recurring phenomenon

UCP1 expression is generally accepted to be highly cell-specific, restricted to brown [1] and beige (e.g. [2]) adipocytes. Nevertheless, the presence of UCP1 mRNA and/or UCP1 protein has repeatedly been reported in tissues other than brown and beige fat: the liver of newborn rats [19]; the skeletal muscle of mice chronically treated with a β_3_-adrenergic agonist [20,21]; the longitudinal muscle layer of peristaltic organs, such as the intestine and uterus [22]; rat and mouse thymocytes [23,24]; the mouse brain cortex [25]; neurons in the central and peripheral nervous system of hibernators [26]; the mouse adrenal gland [27] and the kidney, which is the focus of our current study [[5], [6], [7], [8], [9], [10], [11], [12],^28^]. However, these reports of ectopic UCP1 expression have either not been robustly confirmed or have been conclusively disproven. Thus, the list of tissues with suggested but disproven UCP1 presence – skeletal muscle [29], uterus [30] and thymus [15] – now also includes the kidney, as we demonstrate here no evidence for the presence of UCP1 in the kidney.

### 4.2 UCP1 reporter mouse models – what do they reveal about UCP1 expression in the kidney?

The main data shown here result exclusively from immunological detection of UCP1 protein by antibodies. However, a number of indications of the presence of UCP1 in the kidney result from so-called UCP1-reporter mouse models. Reporter mice are genetically engineered models designed to quantify the expression of a gene of interest, monitor its regulation, and, most relevant to this discussion, identify and track the cells that express the gene. In these mice, the promoter of the gene of interest drives the expression of a reporter, typically a fluorescent protein, intended to reflect the gene's genuine expression. This raises the question why these models may yield misleading results.

The development of at least eight different Ucp1 reporter mouse lines [3,4,[31], [32], [33], [34], [35], [36]] highlights the strong scientific interest in systematically profiling Ucp1 gene expression. These lines can be broadly classified either as conditional reporter mice (in which promoter-driven Cre recombinase activates reporter expression) or knock-in reporter mice (in which the expression of the reporter molecule is under direct control of the promoter). Among these models, the most widely used is the conditional Ucp1-Cre line, in which Cre recombinase is constitutively expressed under the control of the Ucp1 promoter, located in a bacterial artificial chromosome (BAC) [4] integrated into chromosome 1 [37]. When crossed with lines containing integrated reporters (e.g., the NuTRAP line), the resulting reporter expression marks cells that have expressed Cre at any given time, reflecting Ucp1 promoter activity in those cells. The observed high expression of the reporter in the kidney of this line [[5], [6], [7]] strongly suggests that UCP1 is expressed in the kidney. However, in this model, Cre/reporter expression reflects not only ongoing Ucp1 promoter activity but may also reflect any prior activity of the Ucp1 promoter. This could be the reason for the positive outcome.

*Ongoing* Ucp1 promoter activity has been monitored in the inducible Ucp1-CreERT2 mouse model [3]. Following tamoxifen treatment, Ucp1-CreERT2 mice displayed reporter expression in the kidney [7], reflecting ongoing Ucp1 promoter activity and again suggesting that UCP1 is expressed in the adult mouse kidney (although the expression levels were much lower than in the constitutive Ucp1-Cre line). Also this finding contrasts with our observation of no detectable UCP1 in the kidney of adult mice. These contrasting results suggest that the activity of the Ucp1 promoter in the kidney of these reporter mice does not reflect genuine Ucp1 promoter activity in the adult mouse kidney. This also underscores the need for caution when using Ucp1-Cre mice as a genetic tool to investigate gene function in brown and beige adipose tissues.

The Ucp1-iCre mouse line, generated by targeting a Cre cassette directly downstream of the Ucp1 stop codon, was developed as a potentially more reliable tool for lineage tracing and gene manipulation in brown and beige adipose tissues [32]. In these mice, sparse Cre activity is detected in the kidney [28], although at much lower levels and intensity than in the conventional Ucp1-Cre line. A similar pattern is observed in the ventromedial hypothalamus, choroid plexus, adrenal glands, ovary, and testis. Notably, Ucp1-iCre males exhibit a high frequency of Cre-mediated germline recombination [28]. Given the remarkably high reporter (and thus Cre) expression observed in the testis of Ucp1-Cre mice [6], unintended recombination in male gametes may also occur in this model. If that is the case, Cre/reporter expression may occur in cells that do not genuinely express UCP1. Further studies are needed to determine whether this takes place.

In knock-in reporter models [3,31,[33], [34], [35], [36]], ectopic Ucp1 expression has not been explicitly examined. Investigating whether such expression occurs in these models could help explain the ‘leaky’ Ucp1 expression observed in Cre-based Ucp1 reporter models.

### 4.3 (Re)evaluating the role of UCP1 in kidney function

The reported expression of UCP1 in the kidney [[8], [9], [10], [11], [12]] has led to the attribution of several significant functions to UCP1 in kidney regulation. One proposed function is the UCP1-dependent induction of a catabolic state known as “tumour slimming,” characterized by suppressed tumour progression [9,10]. UCP1 ablation has been shown to increase oxidative stress in the kidneys, exacerbating ischemia- or cisplatin-induced acute kidney injury (AKI) in mice [8]. Conversely, virally-mediated UCP1 overexpression in the kidneys protected against AKI, probably by reducing oxidative stress [8] or alleviating lipid accumulation [11]. Additionally, elevated UCP1 levels have been reported to reduce oxidative stress by stabilizing SIRT3, ultimately alleviating renal interstitial fibrosis in chronic kidney disease [12].

The strong correlation between these pathophysiological conditions and apparent UCP1 levels in the kidney, along with the significant effects of UCP1 ablation [8] or UCP1 overexpression [8,11,12], apparently supports the notion that UCP1 is expressed in the kidney. However, this is difficult to reconcile with our findings, as we convincingly demonstrate that UCP1 is not expressed in the kidney. One possibility is that UCP1 expression is induced under the pathophysiological conditions studied. We find this unlikely. A more plausible explanation is that kidney function in UCP1–KO mice is affected by an indirect mechanism, while UCP1 (over)expression in the kidney could still influence kidney function significantly, even if it is not naturally present in the organ.

### 4.4 Experimental design in antibody-based studies

To detect protein expression, many studies rely on antibody-based methods such as immunoblotting and immunostaining. However, two major issues can compromise the reliability of these experiments: failure of an antibody to recognize the intended target, and antibody binding to additional proteins (off-target binding). A common approach to address these concerns is testing antibodies using samples from knockout (KO) cell lines or mice.

Our present study underscores the importance of validating antibodies using knockout samples. Using this approach, we tested four different UCP1 antibodies. All recognized UCP1, but two antibodies (C10 and ab10983) also showed immunopositivity in the kidney. That the immunopositivity observed in the kidney with these two antibodies was *not* due to binding to UCP1 itself but instead to another protein could only be concluded because the same immunopositivity was observed in sections from UCP1–KO mice.

This approach also revealed that two other antibodies (E9Z2V and EPR20381) are markedly specific. These antibodies distinguished brown adipocytes from white adipocytes and wild-type brown adipocytes from those of UCP1–KO mice, with no cross-reactivity in the kidney. The Cell Signaling antibody E9Z2V has also been extensively validated by the company across multiple tissues (although not in the kidney), whereas the Abcam antibody EPR20381 requires further validation in other tissues.

In conclusion, based on these findings, we would recommend the Cell Signaling E9Z2V antibody as the primary choice for UCP1 detection, with the Abcam EPR20381 antibody serving as a reliable alternative.

## CRediT authorship contribution statement

**Celso Pereira Batista Sousa-Filho:** Writing – review & editing, Investigation, Formal analysis. **Natasa Petrovic:** Writing – review & editing, Writing – original draft, Supervision, Project administration, Methodology, Investigation, Funding acquisition, Formal analysis, Conceptualization.

## Declaration of competing interest

The authors declare no conflicts of interest related to the contents of this article.

## Data Availability

Data will be made available on request.
