# Supplementary material for: No UCP1 in the kidney
Source: Mol Metab. 2025 Mar 20;95:102127. doi: 10.1016/j.molmet.2025.102127 (PMC11995138; doi:10.1016/j.molmet.2025.102127)
Supplement: Multimedia component 1 [file mmc1.pdf]

Supplementary data

No UCP1 in the kidney

Celso Pereira Batista Sousa-Filho[1], Natasa Petrovic[1*]

Detailed experimental procedures

Immunohistochemistry

The right kidney, along with the perirenal brown adipose tissue (perirenal BAT), was immersion-fixed in 4 % alcoholic formaldehyde (4 % formaldehyde in ethanol) for 24 hours. It was then dehydrated and embedded in paraffin using a standard procedure [1]. Tissues were sectioned using a standard microtome (Leica RM2255, Leica Microsystems). The sections, each 5 μm thick, were mounted on SuperFrost® Plus adhesion slides (VWR International bvba, Leuven, Belgium). Subsequently, the sections were deparaffinized and rehydrated. To unmask antigenicity, the deparaffinized and rehydrated slides were boiled in citrate buffer (10 mM sodium citrate, pH 6) in a water bath for 30 minutes. After boiling, they were cooled on the benchtop for another 30 minutes. To block autofluorescence, sections were placed in a humid chamber with ethanol vapour, incubated in 0.3% Sudan Black B (Sigma-Aldrich, 199664) in 70% ethanol for 30 minutes at room temperature, and then rinsed with PBS.

In all experiments, antibodies from the same host species (rabbit) were used, necessitating sequential multiplex staining (as described in [2]). Tyramide signal amplification (TSA) kits (Thermo Fisher Scientific, B40943, B40944 and/or B40931) were used in this protocol. To quench endogenous peroxidase activity, slides were placed in a standard humid chamber (with water vapour) and incubated with 3 % hydrogen peroxide solution (Component C2 from the kit) for 1 hour at room temperature. After rinsing, they were incubated in blocking solution (3% BSA in PBS) for 2 hours at room temperature. For the primary antibody incubation, the primary antibodies were diluted in 1 % BSA in PBS. A volume of ~30 μl was pipetted onto each tissue section, and they were then incubated for 24 hours at 4 °C in a humid chamber. Negative

controls, incubated with 1 % BSA in PBS without primary antibody, were included to assess autofluorescence and nonspecific binding.

For clarity, we have described below the procedure for each figure, even though this is somewhat repetitive.

Figures 1 and 2: Antibodies used: UCP1 – rabbit polyclonal raised against C-terminal decapeptide (custom-made, C10), diluted 1:1000; perilipin – rabbit monoclonal (Cell Signaling Technology, 4661S), diluted 1:500; Secondary antibody – goat anti-rabbit labelled with Alexa Fluor 594 (Molecular Probes, A11037), diluted 1:250.

The slides were initially incubated overnight with the UCP1 C10 antibody. Following the C10 antibody incubation, the slides were washed with PBS for 1 h. Then, the slides were incubated with a poly-HRP-conjugated secondary antibody (Component B from the kit (B40943)) for 1 h at room temperature. After this, the slides were washed with PBS for 1 h. Next, the slides were incubated with a tyramide working solution (labelled with Alexa Fluor 488) for 10 minutes, following the instructions provided in the kit. After this step, the slides were not exposed to direct light (whenever possible, the slides were kept in the dark). Following the incubation, the slides were again washed with PBS. To confirm successful staining, the slides were examined under an epifluorescence microscope. The tyramide fluorescence label is covalently bound to the proteins in the sample and, it is also thermally stable. This allowed for the subsequent removal of both the primary and secondary antibodies from the sample by boiling the slides in a citrate buffer. The slides were boiled in the citrate buffer (10 mM sodium citrate, pH 6) in a water bath for 30 minutes. After boiling, the slides were allowed to cool on the benchtop for 30 minutes.

After cooling, the samples were incubated with BSA solution for 30 min. Following this, the samples were incubated with perilipin antibody. After the perilipin antibody incubation, the

slides were washed with PBS for 1 h and then incubated with secondary antibody diluted in 1 % BSA in PBS; a volume of ~30 µl was pipetted onto each tissue section, followed by a 2-hour incubation at room temperature in a humid chamber. After secondary antibody incubation, sections were washed with PBS for 1 h.

To stain nuclei, the sections were incubated in 1 µg/ml Hoechst 33258 (Sigma-Aldrich, 861405) for 10 min, washed with PBS for 30 min and mounted with ProGold® antifade reagent (Molecular Probes, P36934). Sections were analysed in a confocal Zeiss LSM 780 microscope (Carl Zeiss Micro Imaging).

Figure 3: Antibodies used: UCP1 – rabbit polyclonal (Abcam, ab10983), diluted 1:200; perilipin – rabbit monoclonal (Cell Signaling Technology, 4661S), diluted 1:1000.

The slides were initially incubated overnight with the perilipin antibody. Following the perilipin antibody incubation, the slides were washed with PBS for 1 h. Then, the slides were incubated with a poly-HRP-conjugated secondary antibody (Component B from the kit (B40944)) for 1 h at room temperature. After this, the slides were washed with PBS for 1 h. Next, the slides were incubated with a tyramide working solution (labelled with Alexa Fluor 594) for 10 minutes, following the instructions provided in the kit. After this step, the slides were not exposed to direct light (whenever possible, the slides were kept in the dark). Following the incubation, the slides were again washed with PBS. To confirm successful staining, the slides were examined under an epifluorescence microscope. The slides were then boiled in the citrate buffer (10 mM sodium citrate, pH 6) in a water bath for 30 minutes. After boiling, the slides were allowed to cool on the benchtop for 30 minutes.

After cooling, the samples were incubated with BSA solution for 30 min and then with Streptavidin/Biotin Blocking kit (Invitrogen, R37628) (to block endogenous biotin). Following

this, the samples were incubated with UCP1 ab10983 antibody. Then the slides were washed with PBS for 1 h and incubated with a poly-HRP-conjugated secondary antibody for 1 h at room temperature. After this, the slides were washed with PBS for 1 h and then incubated with a tyramide working solution (Biotin-XX tyramide reagent from the kit (B40931)) for 10 minutes, following the instructions provided in the kit. The slides were washed with PBS for 1 h and then incubated with DyLight™ 488-conjugated streptavidin (Invitrogen, 21832), diluted 1:200 in 1 % BSA in PBS. After this, sections were washed with PBS for 1 h.

To stain nuclei, the sections were incubated in 1 µg/ml Hoechst 33258 (Sigma-Aldrich, 861405) for 10 min, washed with PBS for 30 min and mounted with ProGold® antifade reagent (Molecular Probes, P36934). Sections were analysed in a confocal Zeiss LSM 780 microscope (Carl Zeiss Micro Imaging).

Figure 4B,C: Antibodies used: UCP1 – rabbit monoclonal (Cell Signaling Technology, E9Z2V), diluted 1:200; perilipin – rabbit monoclonal (Cell Signaling Technology, 4661S), diluted 1:500; Secondary antibody – goat anti-rabbit labelled with Alexa Fluor 594 (Molecular Probes, A11037), diluted 1:250.

The slides were initially incubated overnight with the UCP1 E9Z2V antibody. Following primary antibody incubation, the slides were washed with PBS for 1 h. Then, the slides were incubated with a poly-HRP-conjugated secondary antibody (Component B from the kit (B40943)) for 1 h at room temperature. After this, the slides were washed with PBS for 1 h. Next, the slides were incubated with a tyramide working solution (labelled with Alexa Fluor 488) for 10 minutes, following the instructions provided in the kit. After this step, the slides were not exposed to direct light (whenever possible, the slides were kept in the dark). Following the incubation, the slides were again washed with PBS. To confirm successful staining, the slides

were examined under an epifluorescence microscope. The slides were then boiled in the citrate buffer (10 mM sodium citrate, pH 6) in a water bath for 30 minutes. After boiling, the slides were allowed to cool on the benchtop for 30 minutes.

After cooling, the samples were incubated with BSA solution for 30 min. Following this, the samples were incubated with perilipin antibody. After the perilipin antibody incubation, the slides were washed with PBS for 1 h and then incubated with secondary antibody diluted in 1 % BSA in PBS; a volume of ~30 µl was pipetted onto each tissue section, followed by a 2-hour incubation at room temperature in a humid chamber. After secondary antibody incubation, sections were washed with PBS for 1 h.

To stain nuclei, the sections were incubated in 1 µg/ml Hoechst 33258 (Sigma-Aldrich, 861405) for 10 min, washed with PBS for 30 min and mounted with ProGold® antifade reagent (Molecular Probes, P36934). Sections were analysed in a confocal Zeiss LSM 780 microscope (Carl Zeiss Micro Imaging).

Figure S1: Antibodies used: Antibodies used: UCP1 – rabbit polyclonal raised against C-terminal decapeptide (custom-made, C10), diluted 1:500; perilipin – rabbit monoclonal (Cell Signaling Technology, 4661S), diluted 1:1000; Secondary antibody – chickent anti-rabbit labelled with Alexa Fluor 488 (Molecular Probes, A21441), diluted 1:200.

The slides were initially incubated overnight with the perilipin antibody. Following the primary antibody incubation, the slides were washed with PBS for 1 h. Then, the slides were incubated with a poly-HRP-conjugated secondary antibody (Component B from the kit (B40944)) for 1 h at room temperature. After this, the slides were washed with PBS for 1 h. Next, the slides were incubated with a tyramide working solution (labelled with Alexa Fluor 594) for 10 minutes, following the instructions provided in the kit. After this step, the slides were not

exposed to direct light (whenever possible, the slides were kept in the dark). Following the incubation, the slides were again washed with PBS. To confirm successful staining, the slides were examined under an epifluorescence microscope. The slides were then boiled in the citrate buffer (10 mM sodium citrate, pH 6) in a water bath for 30 minutes. After boiling, the slides were allowed to cool on the benchtop for 30 minutes.

After cooling, the samples were incubated with BSA solution for 30 min. Following this, the samples were incubated with UCP1 C10 antibody. After the incubation, the slides were washed with PBS for 1 h and then incubated with secondary antibody diluted in 1 % BSA in PBS; a volume of ~30 μl was pipetted onto each tissue section, followed by a 2-hour incubation at room temperature in a humid chamber. After secondary antibody incubation, sections were washed with PBS for 1 h.

To stain nuclei, the sections were incubated in 1 μg/ml Hoechst 33258 (Sigma-Aldrich, 861405) for 10 min, washed with PBS for 30 min and mounted with ProGold® antifade reagent (Molecular Probes, P36934). Sections were analysed in a confocal Zeiss LSM 780 microscope (Carl Zeiss Micro Imaging).

Figure S2: Antibodies used: UCP1 – rabbit polyclonal (Abcam, ab10983), diluted 1:500; perilipin – rabbit monoclonal (Cell Signaling Technology, 4661S), diluted 1:500.
Secondary antibody – goat anti-rabbit labelled with Alexa Fluor 594 (Molecular Probes, A11037), diluted 1:250.

The slides were initially incubated overnight with the UCP1 ab10983 antibody. Following primary antibody incubation, the slides were washed with PBS for 1 h. Then, the slides were incubated with a poly-HRP-conjugated secondary antibody (Component B from the kit (B40943)) for 1 h at room temperature. After this, the slides were washed with PBS for 1 h. Next,

the slides were incubated with a tyramide working solution (labelled with Alexa Fluor 488) for 10 minutes, following the instructions provided in the kit. After this step, the slides were not exposed to direct light (whenever possible, the slides were kept in the dark). Following the incubation, the slides were again washed with PBS. To confirm successful staining, the slides were examined under an epifluorescence microscope. The slides were then boiled in the citrate buffer (10 mM sodium citrate, pH 6) in a water bath for 30 minutes. After boiling, the slides were allowed to cool on the benchtop for 30 minutes.

After cooling, the samples were incubated with BSA solution for 30 min. Following this, the samples were incubated with perilipin antibody. After the perilipin antibody incubation, the slides were washed with PBS for 1 h and then incubated with secondary antibody diluted in 1 % BSA in PBS; a volume of ~30 µl was pipetted onto each tissue section, followed by a 2-hour incubation at room temperature in a humid chamber. After secondary antibody incubation, sections were washed with PBS for 1 h.

To stain nuclei, the sections were incubated in 1 µg/ml Hoechst 33258 (Sigma-Aldrich, 861405) for 10 min, washed with PBS for 30 min and mounted with ProGold® antifade reagent (Molecular Probes, P36934). Sections were analysed in a confocal Zeiss LSM 780 microscope (Carl Zeiss Micro Imaging).

Figure S3: Antibodies used: UCP1 – rabbit monoclonal (Abcam, EPR20381), diluted 1:350; perilipin – rabbit monoclonal (Cell Signaling Technology, 4661S), diluted 1:500; Secondary antibody – goat anti-rabbit labelled with Alexa Fluor 594 (Molecular Probes, A11037), diluted 1:250.

The slides were initially incubated overnight with the UCP1 EPR20381 antibody. Following primary antibody incubation, the slides were washed with PBS for 1 h. Then, the

slides were incubated with a poly-HRP-conjugated secondary antibody (Component B from the kit (B40943)) for 1 h at room temperature. After this, the slides were washed with PBS for 1 h. Next, the slides were incubated with a tyramide working solution (labelled with Alexa Fluor 488) for 10 minutes, following the instructions provided in the kit. After this step, the slides were not exposed to direct light (whenever possible, the slides were kept in the dark). Following the incubation, the slides were again washed with PBS. To confirm successful staining, the slides were examined under an epifluorescence microscope. The slides were then boiled in the citrate buffer (10 mM sodium citrate, pH 6) in a water bath for 30 minutes. After boiling, the slides were allowed to cool on the benchtop for 30 minutes.

After cooling, the samples were incubated with BSA solution for 30 min. Following this, the samples were incubated with perilipin antibody. After the perilipin antibody incubation, the slides were washed with PBS for 1 h and then incubated with secondary antibody diluted in 1 % BSA in PBS; a volume of ~30 µl was pipetted onto each tissue section, followed by a 2-hour incubation at room temperature in a humid chamber. After secondary antibody incubation, sections were washed with PBS for 1 h.

To stain nuclei, the sections were incubated in 1 µg/ml Hoechst 33258 (Sigma-Aldrich, 861405) for 10 min, washed with PBS for 30 min and mounted with ProGold® antifade reagent (Molecular Probes, P36934). Sections were analysed in a confocal Zeiss LSM 780 microscope (Carl Zeiss Micro Imaging).

Gene expression analysis

RNA isolation and cDNA synthesis: Frozen tissues were homogenized in TRI Reagent (T9424; Sigma-Aldrich), and the chloroform-isopropanol method was used to isolate RNA according to

the Sigma-Aldrich TRI Reagent protocol. The RNA concentrations in the samples were measured with a Thermo Scientific NanoDrop One Spectrophotometer. The High-Capacity cDNA Reverse Transcription Kit (Cat. No. 4368814; Applied Biosystems™) was used to reverse transcribe 500 ng of total RNA into cDNA in a total volume of 20 µl. After the reaction was completed, cDNA was diluted 10 times in water.

Real-time qPCR: Ucp1-specific primers were validated before use to ensure good amplification efficiency (90–110%) and specificity (controlled for by melting curve analysis and inclusion of control samples in which the reverse transcriptase had been left out of the reaction). The primer sequences were: forward (5’ - 3’) GGCCTCTACGACTCAGTCCA and reverse (5’ - 3’) TAAGCCGGCTGAGATCTTGT. The primers and PowerUp™ SYBR™ Green Master Mix (Applied Biosystems™, A25742) were premixed in a total volume of 11 µl. The final primer concentration used was 0.3 µM. Two microliters of the diluted cDNA were added to the premixed primer solution to a total volume of 13 µl. All samples were run in triplicate. The Bio-Rad CFX Connect Real-Time system was used to perform the real-time quantitative polymerase chain reaction. The samples were preheated 2 min at 50 °C and 10 min at 95 °C, after which 40 cycles of 15 s at 95 °C and 1 min at 60 °C were run. The real-time qPCR reaction was followed by melting curve analysis.

Ct values were determined by the CFX Manager™ software (Bio-Rad Laboratories) with regression mode. To calculate relative changes in Ucp1 mRNA abundance, the Ct values were antilog-transformed (2^{-Ct}).

[1] Cinti, S., Zingaretti, M.C., Cancello, R., Ceresi, E., Ferrara, P., 2001. Morphologic techniques for the study of brown adipose tissue and white adipose tissue. Methods Mol Biol 155:21-51.
[2] Toth, Z.E., Mezey, E., 2007. Simultaneous visualization of multiple antigens with tyramide signal amplification using antibodies from the same species. J Histochem Cytochem 55(6):545-554.

Figure S1

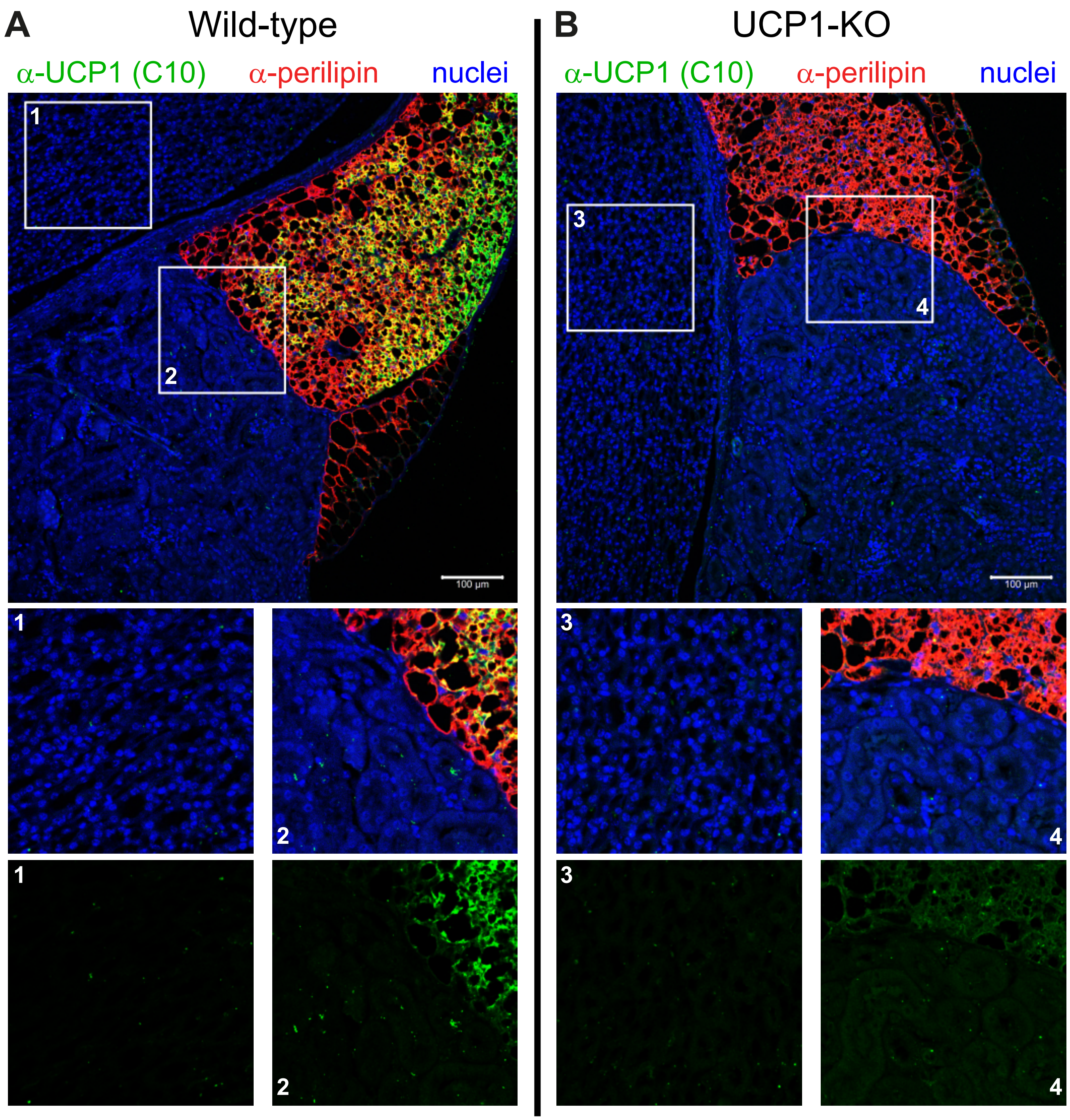

α-UCP1 - rabbit polyclonal antibody (C10), custom-made, raised against C-terminal decapeptide

Figure S1. UCP1 antibody C10 shows no observable cross-reactivity when using a fluorescently labelled secondary antibody for signal detection. (A-B) Representative confocal images of the transversal section of the kidney, also showing adjacent perirenal BAT, obtained with the UCP1 C10 antibody. Wild-type (A) and UCP1-KO (B) male mice, acclimated to room temperature and approximately two months old, were analysed, as in Fig. 2. UCP1 – green; perilipin (an adipocyte identity marker) – red; nuclei – blue. Magnified insets provide better visualization of the kidney papilla (insets 1 and 3) and perirenal BAT and the kidney parenchyma (insets 2 and 4). Scale bars, 100 µm.

In contrast to Figure 1 and Figure 2, the UCP1 antibody-derived signal here was detected using a fluorescently labelled secondary antibody. Staining in all cases involved tyramide signal amplification (TSA), which allows for co-staining with two antibodies derived from the same

species (here, the two rabbit-derived antibodies) [1] (see Detailed experimental procedures in the Supplementary data (above)). Due to signal amplification, TSA is a highly sensitive detection method. This method requires that the two rabbit-derived antibodies are applied sequentially. In Figure 1 and Figure 2, the UCP1 antibody was applied first and visualized using highly sensitive TSA, while the perilipin antibody was applied thereafter and visualized with a fluorescently labelled secondary antibody.

To determine whether signal amplification caused the observed nonspecific signal(s) in Figure 1 and Figure 2, we reversed the order of antibody application: the perilipin antibody was visualized with TSA, followed by visualization of the UCP1 antibody with the fluorescently labelled secondary antibody. Importantly, as shown above (Figure S1), this reversed protocol yielded a completely different result. The only immunopositivity observed was in the perirenal BAT of wild-type mice (main Figure S1A and magnified inset 2), with essentially no staining in the kidney (main Figure S1A-B and magnified insets 1 and 3) or in the perirenal BAT of UCP1-KO mice (main Figure S1B and magnified inset 4).

[1] Toth, Z.E., Mezey, E., 2007. Simultaneous visualization of multiple antigens with tyramide signal amplification using antibodies from the same species. J Histochem Cytochem 55(6):545-554.

Figure S2

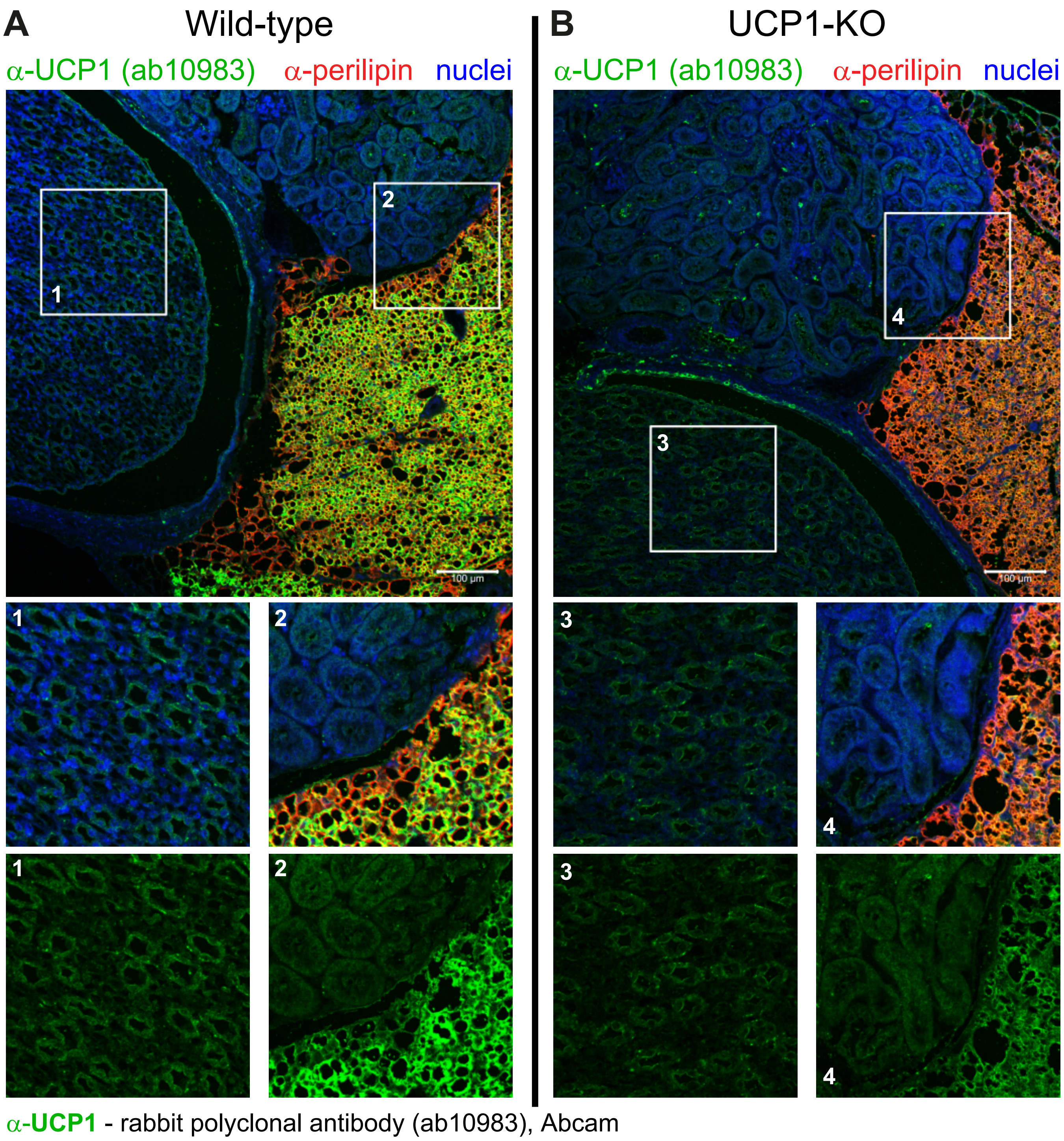

Figure S2. UCP1 antibody ab10983 shows cross-reactivity even when the signal is detected without extensive amplification. (A-B) Representative confocal images of the transversal section of the kidney, also showing adjacent perirenal BAT, obtained using the rabbit polyclonal antibody from Abcam (ab10983). Wild-type (A) and UCP1-KO (B) male mice, acclimated to room temperature and approximately two months old, were analysed as in Figure 2. UCP1 – green; perilipin (an adipocyte identity marker) – red; nuclei – blue. Magnified insets provide better visualization of the kidney papilla (insets 1 and 3) and the kidney parenchyma and perirenal BAT (insets 2 and 4). Scale bars, 100 μm.

Here, the same protocol as in Figure 1 and Figure 2 was applied: the UCP1 antibody was applied first and visualized using highly sensitive tyramide signal amplification (TSA), followed by the perilipin antibody, which was detected with a fluorescently labelled secondary antibody. The strongest and most distinct signal was observed in the perirenal BAT of wild-type mice (green) (Figure S2A and magnified inset 2), indicating its highest affinity for UCP1. Weaker but still distinguishable staining was observed in the kidney (both parenchyma and papilla), with a pattern unaffected by genotype (Figure S2A-B). The observed staining closely resembled previous results obtained with this antibody [1-5]. To better visualize the immunopositive structures in the kidney, we modified the staining protocol to incorporate further amplification of the UCP1 antibody-derived signal (presented in Figure 3). For details, see Detailed experimental procedures in the Supplementary data (above). To our understanding, the detection method (in terms of sensitivity) employed in Figure 3 should be similar to the methods used in studies observing similar structures with this antibody [1-5] (based on the available, albeit incomplete, method descriptions).

[1] Jia, P., Wu, X., Pan, T., Xu, S., Hu, J., Ding, X., 2019. Uncoupling protein 1 inhibits mitochondrial reactive oxygen species generation and alleviates acute kidney injury. EBioMedicine 49:331-340.

[2] Xiao, W., Xiong, Z., Xiong, W., Yuan, C., Xiao, H., Ruan, H., et al., 2019. Melatonin/PGC1A/UCP1 promotes tumor slimming and represses tumor progression by initiating autophagy and lipid browning. J Pineal Res 67(4):e12607.

[3] Xiong, Z., Xiao, W., Bao, L., Xiong, W., Xiao, H., Qu, Y., et al., 2019. Tumor Cell "Slimming" Regulates Tumor Progression through PLCL1/UCP1-Mediated Lipid Browning. Adv Sci (Weinh) 6(10):1801862.

[4] Xiong, W., Xiong, Z., Song, A., Lei, C., Ye, C., Zhang, C., 2021. Relieving lipid accumulation through UCP1 suppresses the progression of acute kidney injury by promoting the AMPK/ULK1/autophagy pathway. Theranostics 11(10):4637-4654.

[5] Xiong, W., Xiong, Z., Song, A., Lei, C., Ye, C., Su, H., et al., 2023. UCP1 alleviates renal interstitial fibrosis progression through oxidative stress pathway mediated by SIRT3 protein stability. J Transl Med 21(1):521.

Figure S3

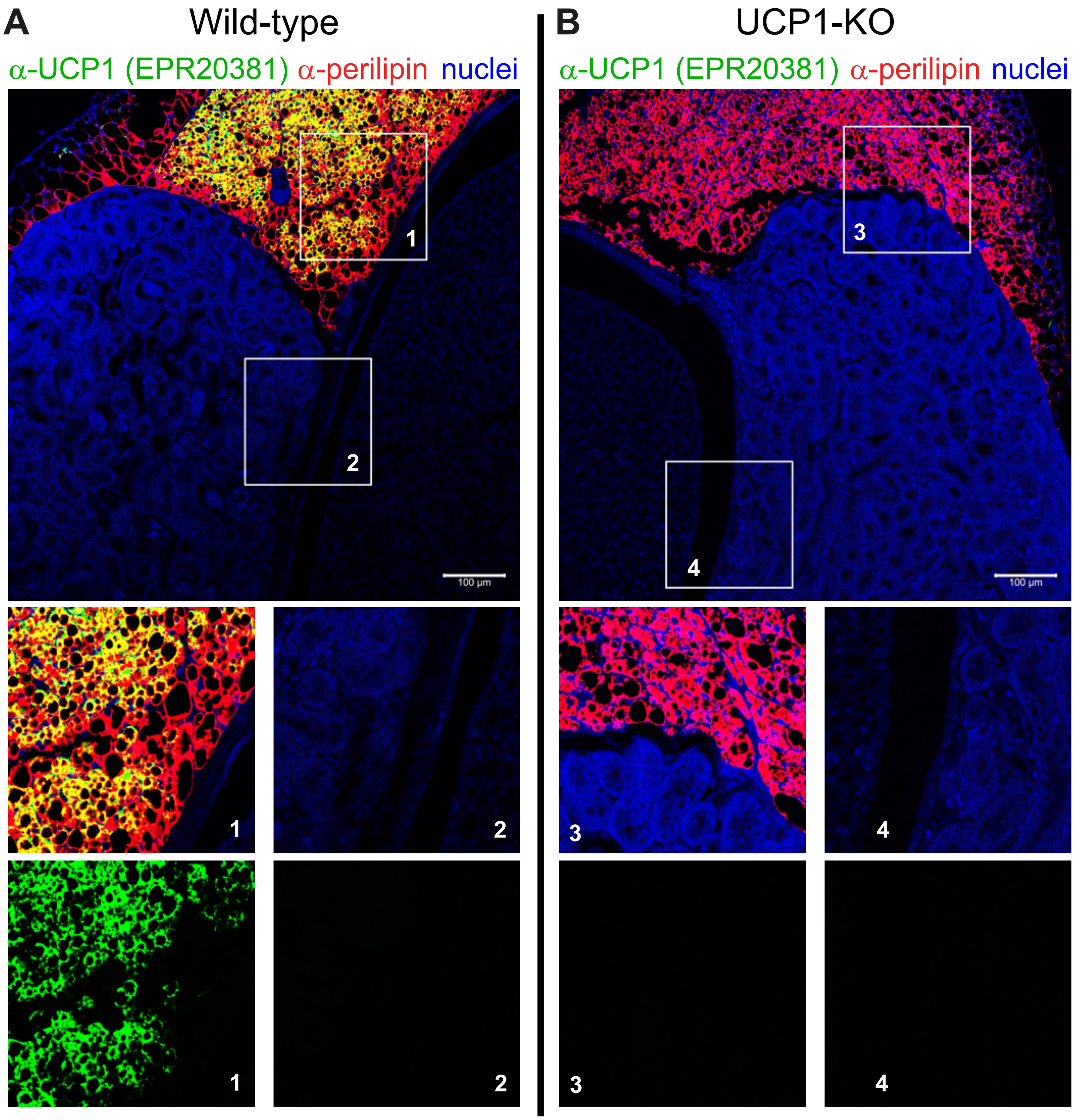

α-**UCP1** - rabbit monoclonal antibody (EPR20381), Abcam

Figure S3. UCP1 is not expressed in the kidney. (A-B) Representative confocal images of the transversal section of the kidney, also showing adjacent perirenal BAT, obtained using the rabbit monoclonal antibody (EPR20381) from Abcam (ab209483). Wild-type (A) and UCP1-KO (B) male mice, acclimated to room temperature and approximately two months old, were analysed as in Figure 2. UCP1 – green; perilipin (an adipocyte identity marker) – red; nuclei – blue. Note the complete absence of immunoreactivity to this UCP1 antibody in both the perirenal BAT and the kidney of UCP1-deficient mice (B). Magnified insets provide better visualization of perirenal BAT and the kidney parenchyma (insets 1 and 3), as well as the kidney parenchyma and papilla (insets 2 and 4). Scale bars, 100 µm.
